# A Curcumin-Decorated Nanozyme with ROS Scavenging and Anti-Inflammatory Properties for Neuroprotection

**DOI:** 10.3390/nano14050389

**Published:** 2024-02-20

**Authors:** Feng Gao, Wenyu Liang, Qixin Chen, Bairu Chen, Yuchen Liu, Zhibo Liu, Xu Xu, Rongrong Zhu, Liming Cheng

**Affiliations:** 1 Department of Orthopedics, Tongji Hospital Affiliated to Tongji University, School of Medicine, Tongji University, Shanghai 200331, China; 2 Key Laboratory of Spine and Spinal Cord Injury Repair and Regeneration of Ministry of Education, School of Life Science and Technology, Tongji University, Shanghai 200065, China; 3 Frontier Science Center for Stem Cell Research, Tongji University, Shanghai 200065, China

**Keywords:** nanozyme, ROS scavenging, anti-inflammatory, neuroprotective

## Abstract

Disordered reactive oxygen/nitrogen species are a common occurrence in various diseases, which usually cause cellular oxidative damage and inflammation. Despite the wide range of applications for biomimetic nanoparticles with antioxidant or anti-inflammatory properties, designs that seamlessly integrate these two abilities with a synergistic effect in a simple manner are seldom reported. In this study, we developed a novel PEI-Mn composite nanoparticle (PM NP) using a chelation method, and the curcumin was loaded onto PM NPs via metal–phenol coordination to form PEI-Mn@curcumin nanoparticles (PMC NPs). PMC NPs possessed excellent dispersibility and cytocompatibility, was engineered to serve as an effective nanozyme, and exhibited specific SOD-like and CAT-like activities. In addition, the incorporation of curcumin granted PMC NPs the ability to effectively suppress the expression of inflammatory cytokines in microglia induced by LPS. As curcumin also has antioxidant properties, it further amplified the synergistic efficiency of ROS scavenging. Significantly, PMC NPs effectively scavenged ROS triggered by H_2_O_2_ in SIM-A9 microglia cells and Neuro-2a cells. PMC NPs also considerably mitigated DNA and lipid oxidation in Neuro-2a cells and demonstrated an increase in cell viability under various H_2_O_2_ concentrations. These properties suggest that PMC NPs have significant potential in addressing excessive ROS and inflammation related to neural diseases.

## 1. Introduction

Reactive oxygen species (ROS) act as pivotal signaling cues in normal physiological conditions, playing crucial roles in signal transduction and immune regulation [1,2,3,4]. Disrupted ROS generation has been observed in the pathology of many diseases, particularly in central nervous system (CNS) conditions [5,6,7], such as spinal cord injury (SCI), traumatic brain injury (TBI), Alzheimer’s disease (AD), and Parkinson’s disease (PD). Excessive ROS can induce serious oxidative stress, resulting in DNA, lipid, and protein oxidative damage [8,9]. This also triggers widespread secondary injuries like axonal demyelination and neuronal cell necrosis [10]. Concurrently, an inflammatory response mediated by microglia gets activated due to excessive ROS [11]. As resident immune cells in the CNS, microglia oscillate between two states in neuroinflammation; the pro-inflammatory microglia secrete numerous inflammatory cytokines and chemokines, intensifying inflammation, while anti-inflammatory microglia foster tissue regeneration [12,13,14]. Hence, managing the state of microglia could be beneficial in controlling the inflammatory response in CNS diseases.

The balance of endogenous ROS is maintained by an antioxidant protective system comprising oxidase (OXD), peroxidase (POD), superoxide dismutase (SOD), and catalase (CAT). Notably, SOD catalyzes the conversion of superoxide anion (•O_2_) into H_2_O_2_ and oxygen (O_2_), while CAT converts H_2_O_2_ into H_2_O and O_2_. As such, enzyme-based therapy has emerged as an alternative for diseases related to ROS imbalance [15,16]. However, the use of natural enzymes is restricted due to their poor stability, single enzyme activity, and limited reusability [17]. In response to the adverse effects of ROS, nanomaterials with enzyme-mimetic abilities have shown potential as future therapies. They offer higher catalytic efficiency in physiological environments and greater design flexibility, along with the ability to integrate with multiple enzymes [18,19,20,21]. Despite these promising developments, challenges remain, including the complexity of synthetic processes and the composition of the nanomaterial [22]. Therefore, simple synthesis and rational design of multifunctional enzyme-mimetic nanomaterials are essential.

Curcumin, a polyphenolic chemical, has been extensively studied for its anti-inflammatory and antioxidant properties [23,24]. Numerous studies have reported its neuroprotective effects on the CNS and peripheral nervous system (PNS) diseases [25]. Curcumin can inhibit inflammatory response in SCI by repolarizing microglia and macrophages [26,27]. Additionally, it has been reported to activate the Nrf2/HO-1 signaling pathway and scavenge free radicals in spinal cord injuries [28]. However, its poor water solubility and low bioavailability limit its use in treating various diseases [29].

In this study, a novel Mn-based nanozyme adorned with curcumin was synthesized for antioxidation and anti-inflammation. The fabricated PMC NPs displayed excellent dispersity and promising biocompatibility. They demonstrated significant antioxidant enzyme-mimicking activity, specifically in SOD-like and CAT-like activities under physiological conditions. The formation process and curcumin-loading mechanism were explored, and the influence of curcumin on the enzyme-mimicking activity of PMC NPs was investigated. When applied to H_2_O_2_-induced SIM-A9 microglia, PMC NPs displayed superior intracellular ROS scavenging ability and effectively inhibited the inflammatory response triggered by LPS. They also reduced the oxidative damage in DNA and lipids in the Neuro-2a cell line through ROS scavenging, ensuring cell viability under oxidative conditions. Consequently, with high biocompatibility, these easily synthesized PMC NPs could effectively alleviate oxidative stress and reduce neuroinflammation, demonstrating potential as novel therapeutic agents for neural diseases.

## 2. Materials and Methods

### 2.1. Reagents and Chemicals

Polyethyleneimine (PEI, M.W. 10,000, 99%), manganese chloride tetrahydrate (MnCl_2_·4H_2_O), and 3,3′,5,5’-Tetramethylbenzidine (TMB) were purchased from Aladdin Reagent Co., Ltd. (Shanghai, China). Hydrogen peroxide (H_2_O_2_, 30%), hydrochloric acid (HCl), sodium acetate (NaAc), acetic acid (HAc), and ethanol (C_2_H_5_OH) were obtained from Sinopharm Chemical Reagent Co., Ltd. (Shanghai, China). Titanium (IV) sulfate (Ti(SO_4_)_2_) was provided by Macklin (Shanghai, China). All the chemicals were of analytical grade and used directly without further purification.

Curcumin was purchased from MedChemExpress (Monmouth Junction, NJ, USA). Lipopolysaccharide (LPS) was provided by Sigma Aldrich (St. Louis, MO, USA). TNF-α (#ab1793), iNOS (#ab178945) and IL-10 (#ab9969) were obtained from Abcam (Cambridge, MA, USA). H2AX (#10856-1-AP) and β-actin (#66009-1-Ig) were obtained from Proteintech (Wuhan, China). Calcein AM, Propidium Iodide, the Total Superoxide Dismutase Assay Kit with WST-8, the Reactive Oxygen Species Assay Kit, and the Lipid Peroxidation MDA Assay Kit were obtained from Beyotime (Shanghai, China). The ABTS Free Radical Scavenging Capacity Assay Kit, DPPH Free Radical Scavenging Capacity Assay Kit, and CCK-8 Cell Proliferation and Cytotoxicity Assay Kit were purchased from Solarbio (Beijing, China).

### 2.2. Synthesis of PM and PMC NPs

PMC NPs were synthesized by facile stirring at room temperature. Briefly, 50 mg of PEI was dissolved in 100 mL of deionized water, and 200 mg of MnCl_2_·4H_2_O was dissolved in 4 mL of deionized water, by slowly adding it drop by drop while stirring. After 30 min, the PM NPs were collected using a centrifuge and thoroughly washed with deionized water and ethanol. For the synthesis of PMC NPs, 10 mg of PM NPs was dispersed evenly in 50 mL of absolute ethanol using an ultrasonicator. Then, 10 mg of curcumin was added and mixed for 24 h. The PMC NPs were thoroughly washed with ethanol and collected using a centrifuge.

### 2.3. Characterization

The morphology of samples was observed by scanning electron microscope (ZEISS Gemini SEM 300, ZEISS, Jena, Germany) under the acceleration voltage of 5.0 kV. Transmission electron microscopy (TEM) investigations were performed by JEOL JEM-F200 (Tokyo, Japan). The phase and crystal structure of samples were analyzed by X-ray diffractometer (XRD, Rigaku Ultima IV, Los Angeles, CA, USA). Fourier transform infrared (FTIR) transmission spectra of samples were obtained on a Thermo Scientific Nicolet iS20 spectrophotometer (Waltham, MA, USA). The chemical compositions were measured via X-ray photoelectron spectroscopy (XPS, Thermo Scientific K-Alpha, Waltham, MA, USA). The hydrodynamic diameter, zeta potential, and polydisperse index of the material were analyzed by a nanoparticle size analyzer (Malvern Panalytical, Malvern, UK). The absorption spectrum of the material was examined on a UV–visible spectrometer (Shimadzu, Kyoto, Japan).

### 2.4. Enzyme-Mimicking ROS Scavenging Ability

#### 2.4.1. Total ROS Scavenging Ability

The ABTS can be oxidized to stable blue-green cationic ABTS radicals, with a maximum absorption peak at 405 nm. The samples possess antioxidant capacity that can reverse this color change; so, the antioxidant capacity of PM and PMC NPs (0–80 μg/mL) were evaluated by the absorbance range at 375–425 nm by the UV–vis spectrophotometer. Similarly, DPPH free radical is a stable nitrogen-centered free radical. Its alcohol solution is purple in color and has strong absorption at 515 nm, which can be used to evaluate the antioxidant capacity. PM and PMC NPs with various concentrations were added to the DPPH solution, and the UV–vis absorption range of 350–700 nm was observed.

#### 2.4.2. Enzyme-Mimicking Activity Measurements

To further investigate the antioxidant capacity, the SOD, CAT, POD, and XOD-like activities of PM and PMC NPs were studied. The SOD-like activities of PM and PMC NPs were assessed by the Total Superoxide Dismutase Assay Kit (Beyotime, Shanghai, China) with Tetrazolium salt-2-[2-methoxy-4-nitrophenyl]-3-[4-nitrophenyl]-5-[2,4-disulfophenyl]-2H-tetrazolium (WST-8). After incubation at 37 °C for 15 min, the UV–vis spectra range 400–500 nm was measured.

For the CAT-like activity test, Ti(SO_4_)_2_ chromogenic tests were used to evaluate the H_2_O_2_ consumption of catalysts. PM and PMC NPs with gradient concentrations (0–80 μg/mL) were incubated in PBS buffers with H_2_O_2_ (5 mM) at 37 °C for 24 h. Then, 20 μL of 1% Ti(SO_4_)_2_ was added to 100 μL of the above mixture solution. After incubating for 5 min, the absorbance at 405 nm of the solution was measured. PBS buffer with and without H_2_O_2_ was set as the positive control and negative control, respectively. H_2_O_2_ scavenging efficiency was calculated by the following formula:

H_2_O_2_
*scavenging efficiency* (%) = (*A_o_* − *A_n_*)/(*A_p_* − *A_n_*) × 100%

where *A_o_*, *A_n_*, and *A_p_* are the absorbance of the treated samples, negative control, and positive control, respectively.

The OXD-like activity of PM or PMC NPs was evaluated using TMB. Briefly, sample solutions (100 μL, 0–80 μg/mL) and TMB (100 μL, 20 mM) were mixed in PBS buffers (pH = 7.4, 0.1 M) with a total volume of 1 mL. After being incubated for 30 min at 37 °C, the absorbance spectra of ox-TMB (500–800 nm) were recorded. The POD-like activity of samples was similar to that of the OXD-like activity with the addition of H_2_O_2_ (100 μL, 1 mM).

### 2.5. Cytotoxicity Test and Hemolysis Assay

The cytotoxicity of PM and PMC NPs (0–80 μg/mL) was evaluated using the CCK-8 assay and Calcein AM/PI cell staining in mouse neuroblastoma Neuro-2a, mouse fibroblast NIH-3T3, and microglia SIM-A9 cell lines. After incubating the cells with the materials for 24 h, the CCK-8 reagent was added and incubated with the cells for 2 h, and the OD450 was recorded to evaluate cell viability. Similarly, after 24 h, the cells were stained with calcein AM/PI for 30 min, and live cells (green) or dead cells (red) were observed under a fluorescent microscope.

For hemocompatibility assessment, a hemolysis assay was performed using blood samples collected from healthy mice and stabilized with sodium citrate. Whole blood (1 mL) was diluted with 1.25 mL of normal saline. PM and PMC NPs (0–80 μg/mL, 10 mL) dispersed in normal saline were incubated at 37 °C for 30 min, respectively. Then, 200 μL of the diluted blood solution was added, and the mixture was incubated at 37 °C for 60 min, followed by centrifugation at 2500 rpm for 5 min. The blood samples diluted with normal saline solution and ultra-water were set as the negative and positive control groups, respectively.

All the supernatant was measured with a multifunctional microplate reader at 545 nm. The hemolysis ratio was calculated by the following formula:

*HR* = (*OD_t_* − *OD_n_*)/(*OD_p_* − *OD_n_*) × 100%

where the *HR* was the hemolysis ratio; *OD_t_* was the absorbance of the samples; *OD_p_* was the absorbance of the positive group; *OD_n_* was the absorbance of the negative group.

### 2.6. Fluorescence Assay of ROS Level

The intracellular ROS levels were evaluated using the DCFH-DA fluorescent probe. SIM-A9 and Neuro-2a cells were seeded in 24-well plates at a cell density of 1 × 10^6^ per well and incubated at 37 °C for 24 h. Then, H_2_O_2_ was added at an experimental concentration of 200 μM to induce the generation of intracellular ROS. After incubating for 12 h, PM and PMC NPs were added to the corresponding wells at a concentration of 10 μg/mL. Following a 24 h incubation, the cells were washed with the serum-free cell culture medium and stained with DCFH-DA for 30 min, followed by another wash with the serum-free cell culture medium. The DCFH-DA fluorescent images of the different groups were captured using a laser confocal microscope.

### 2.7. Immunofluorescence

For the evaluation of the anti-inflammatory effect of PM and PMC NPs, SIM-A9 cells were seeded in 24-well plates with cell climbing sheets at a cell density of 1 × 10^6^ per well and incubated at 37 °C for 24 h. Then, LPS was added at an experimental concentration of 200 ng/mL to induce inflammation. After incubating for 12 h, PM and PMC NPs were added to the corresponding wells at a concentration of 10 μg/mL. Similar to SIM-A9 cells, to assess the antioxidative capability of PM and PMC NPs, Neuro-2a cells were seeded in 24-well plates and processed in the same way as mentioned above for ROS detection.

After treating the cells with the materials for 24 h, they were washed with a PBS solution and fixed in acetone at 4 °C. Then, primary antibodies for TNF-α and iNOS were incubated with the SIM-A9 cells, while H2AX was incubated with the Neuro-2a cells overnight at 4 °C. Fluorescent secondary antibodies were incubated with the cells in the dark at 37 °C for one hour. Nuclei were labeled with DAPI. The samples were observed using a laser confocal microscope, and ImageJ was used for quantitative analysis.

### 2.8. Western Blot

As for Western blot analysis, SIM-A9 cells was cultured in 6-well plate, the inflammation-induced treatment was the same as mentioned above. After the extraction of the protein, sodium dodecyl sulfate polyacrylamide gel electrophoresis was used to separate the protein, and a polyvinylidene fluoride membrane transferred the proteins. The membranes were incubated with IL-10, TNF-α, and iNOS primary antibodies and the corresponding secondary antibody respectively. The targeted protein bands were visualized on X-ray film via the enhanced chemiluminescence reagents. The band gray value was quantified by the ImageJ 2 software.

### 2.9. Statistical Analysis

All experimental data are represented as mean ± standard deviation (SD). One-way analysis of variance (ANOVA) followed by Tukey’s multiple comparison test was used to evaluate the results. The difference between the groups was compared by GraphPad Prism 9 Software. *p* < 0.05 was considered statistically significant.

## 3. Results and Discussion

### 3.1. Synthesis and Characterization of PM and PMC NPs

**Figure 1:**
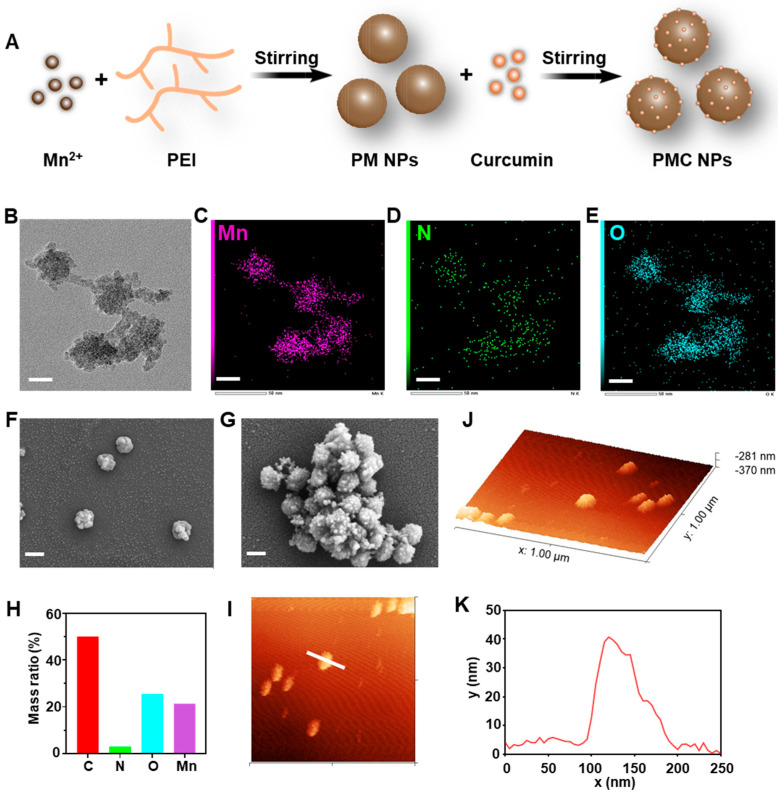
Synthesis and characterization. (**A**) Schematic diagram of PMC NPs’ synthesis process. Transmission electron microscope images (**B**) and EDX element mapping images (**C**–**E**) of PMC NPs (scale bar = 20 nm). Scanning electron microscope images of (**F**) PM NPs and (**G**) PMC NPs (scale bar = 200 nm). (**H**) Element content of PMC NPs. (**I**–**K**) AFM image and the height analysis of PMC NPs.

The PM and PMC NPs were synthesized using a simple method first (Figure 1A). In brief, PM NPs was synthesized by adding MnCl_2_ into the PEI solution dropwise while stirring vigorously. Subsequently, the resulting PM NPs were dispersed in absolute ethanol in the presence of curcumin, and the mixture was incubated overnight at 4 °C to yield the desired PMC NPs. The TEM images of PMC NPs are shown in Figure 1B; it exhibits an irregular spherical morphology with a size of about 50 nm. And element mapping demonstrated the presence and uniform distribution of Mn, N, and O elements in the PMC NPs (Figure 1C–E), with a mass ratio of Mn reaching 21% (Figure 1H). SEM results of PM and PMC NPs showed similar morphology to that of TEM (Figure 1F,G). AFM images further confirmed the successful synthesis of PMC NPs (Figure 1I,J), with the analysis showing a height about 40 nm (Figure 1K).

**Figure 2:**
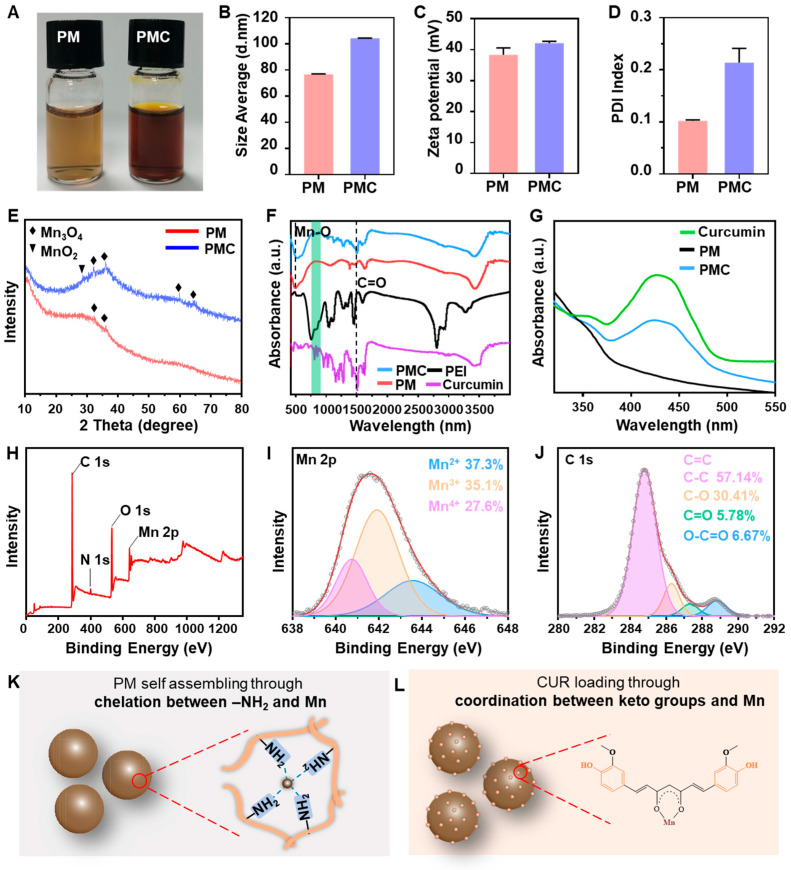
Structure characterization. (**A**) Optical photographs, (**B**–**D**) size, zeta potential, and PDI index by DLS of PM and PMC NPs. (**E**) XRD patterns of PM and PMC NPs. (**F**) FTIR spectrum of PEI, curcumin, PM, and PMC NPs. (Dashed line in the left represented the Mn-O bind appeared in PM and PMC NPs, dashed line in the left represented the C=O bind in curcumin and PMC NPs), (**G**) UV–vis spectrum of curcumin, PM, and PMC NPs. (**H**) XPS spectrum, (**I**) Mn 2p, and (**J**) C 1s high-resolution XPS spectrum of PMC NPs. (The dark cycles and red lines in (**I**,**J**) represented the actual data and fit curves, respectively) (**K**) PM NPs’ synthesis and (**L**) curcumin-loading mechanisms.

The optical photographs of PM and PMC NPs presented in Figure 2A show that PM NPs exhibited a light brown color, while PMC NPs appeared as a deeper brown color after curcumin loading. The hydrodynamic diameters of PM and PMC NPs were measured to be around 80 nm and 100 nm by dynamic light scattering (Figure 2B), respectively. Both NPs had a zeta potential of about 40 mV, making them easily taken up by cells and suggesting potential for nucleic acid loading (Figure 2C). Furthermore, the PDI of both NPs lower than 0.2 in PBS solution indicated their outstanding dispersity, which is beneficial for their utilization in physiological conditions (Figure 2D).

For further structure and component studies, XRD, FITR, UV–vis, and XPS analysis were performed on the materials. As shown in Figure 2E, PM NPs showed a weak crystal structure, while PMC NPs exhibited slight signals of MnO_2_ at a 2θ value of 29.58° and obvious signals of Mn_3_O_4_ at 2θ values of 32.1, 36.06, 59.82, and 64.56° (JCPDS database, card no. 24-0734). Then, the FTIR spectra of PEI, curcumin, PM, and PMC NPs were recorded (Figure 2F). The strong signal of PM and PMC at 500 cm^−1^ could be assigned to the Mn-O band, and the enhanced signal at 604 cm^−1^ in PMC may be attributed to the coordination between Mn and O in the ketone groups of curcumin. In the case of curcumin loaded in PMC NPs, the peak appearing at 1514 cm^−1^ was attributed to the stretch of C=C, and benzene ring peaks appeared at 750–900 cm^−1^. UV–vis analysis revealed that the absorption spectra of PMC NPs contained the absorption peak of curcumin (400–500 nm), which was not present in PM NPs (Figure 2G). After detailed calculation, it was determined that PMC NPs exhibited excellent drug-loading ability, with a curcumin-loading concentration of approximately 49.77 μg/mg (4.977%) at pH 7.4. The XPS data showed characteristic peaks of Mn 2p, O 1s, N 1s, and C 1s in PMC NPs (Figure 2H). The Mn 2p high-resolution XPS spectra of PMC NPs indicates that the valence states of the Mn element were Mn^2+^ (37.3%), Mn^3+^ (35.1%), and Mn^4+^ (27.6%), with binding energies at 643.58, 641.88, and 640.78 eV, respectively (Figure 2I). The C 1s high-resolution XPS spectra could be well fitted by four peaks at 284.78, 286.28, 287.28, and 288.78 eV, corresponding to C=C/C-C (57.14%), C-O (30.41%), C=O (5.78%), and COOH (6.67%), respectively (Figure 2J). All of these data confirmed that the PMC NPs have a stable framework consisting of polyvalent Mn and PEI, making them a suitable carrier for curcumin. Based on the above data, in this design, the PM NPs were formed through a self-assembly process driven by chelation between Mn^2+^ and abundant -NH_2_ in PEI (Figure 2K), while curcumin was loaded through coordination between ketone groups and Mn^2+^ in PMC NPs (Figure 2L).

### 3.2. ROS Scavenging Ability of PMC NPs Intracellular

**Figure 3:**
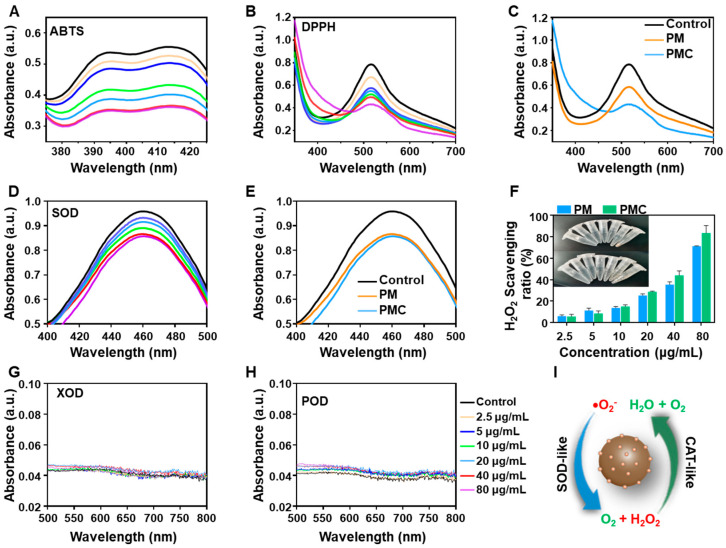
Multiple enzyme-mimicking catalytic abilities. (**A**) The concentration-dependent investigation of ABTS. (**B**) DPPH in the presence of PMC NPs. (**C**) UV–vis spectra of the DPPH eliminated by PM and PMC NPs in same concentration. (**D**) The concentration-dependent investigation of WST-formazan system to determine the SOD-like activity of PMC NPs. (**E**) UV–vis spectra of the SOD-like activity of PM and PMC NPs in same concentration. (**F**) H_2_O_2_ scavenging ratios and oxygen generation catalyzed by PMC NPs in pH = 7.4 PBS buffers. UV–vis spectra of TMB oxidized by PMC NPs without (**G**) and with (**H**) H_2_O_2_ in pH = 7.4 PBS buffers. (**A**,**B**,**D**,**G**,**H**) share the same figure legend in the right of (**H**). (**I**) Schematic presentation of cascade ROS scavenging activities of PMC NPs.

Since Mn-based nanomaterials have been reported to be used as nanozymes for scavenging ROS [30,31,32] and polyphenols such as curcumin are recognized as natural antioxidants that inhibit inflammation, we first studied the total antioxidant ability of PMC NPs for scavenging RONS using 2,2′-azinobis(3-ethylbenzthiazoline-6-sulfonate) (ABTS) and 1-diphenyl-2-picrylhydrazyl (DPPH) assays, respectively. As shown in Figure 3A, PMC NPs exhibited dose-dependent ROS scavenging activity, as the absorbance gradually decreased with higher concentrations of PMC NPs. Meanwhile, the DPPH assay demonstrated the effective scavenging ability of PMC NPs for reactive nitrogen species (RNS) in a dose-dependent manner (Figure 3B). Furthermore, PMC NPs showed better antioxidative activities than PM NPs at the same concentration (Figure 3C), which could be attributed to the coordination of curcumin in PMC NPs.

To better understand the antioxidative activities of PMC NPs, we subsequently evaluated their antioxidant enzyme-mimicking abilities, including SOD-like and CAT-like activities. The SOD-like activity of PMC NPs, which enables the transformation of O^2−^ to H_2_O_2_, was tested using the WST-8 cascade reaction system. As shown in Figure 3D,E, PMC NPs decreased the absorption at 450 nm and exhibited similar efficiency with PM NPs, indicating good SOD-mimicking activity. The CAT-like activity of PM and PMC NPs were evaluated by investigating the decomposition of H_2_O_2_. The results are presented in Figure 3F, showing that both NPs possess CAT-like activity, while PMC NPs are able to eliminate approximately 80% of H_2_O_2_ at a concentration of 80 µg/mL. The generation of O_2_ was also visually observed, with a significant number of bubbles observed in solutions containing PM and PMC NPs in the presence of H_2_O_2_, with the phenomenon being more pronounced in PMC NPs.

The OXD-like and POD-like activities of PMC NPs were also studied using the typical TMB colorimetric assay. As shown in Figure 3G,H, for PMC NPs, negligible absorbances were observed for PMC NPs at various concentrations without or with H_2_O_2_ in pH 7.4 PBS buffers, suggesting that PMC NPs only slightly induced the oxidation of TMB in physiological environments. Based on the above results, PMC NPs specifically demonstrated SOD-like and CAT-like catalytic performance, but not OXD-like and POD-like activities (Figure 3I). These properties can effectively reduce oxidative damage caused by ROS in vitro.

Given the composition and structure of PMC NPs, Mn^3+^/Mn^2+^ redox couples might provide abundant redox reaction sites for the elimination of ROS. In recent studies on metal–phenol coordination nanoparticles, it has been reported that curcumin ligand in the coordination compound can modulate the catalytic activity of metal ions to present SOD-like activity that scavenges O^2−^, while the chelation of metal element can also regulate the electron distribution of the curcumin ligand favoring hydrogen donation to scavenge •OH [33].

### 3.3. Cell Compatibility and Hemocompatibility

**Figure 4:**
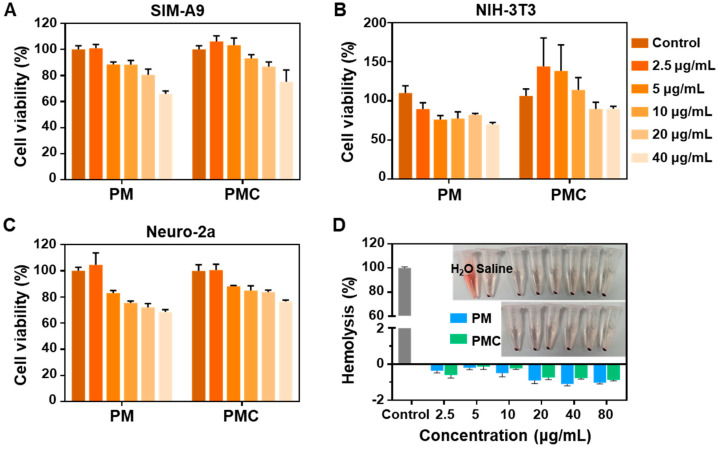
Biosafety assessment of PM and PMC NPs. Cell viability of cells treated with PMC NPs in different concentrations for 24 h (**A**) SIM-A9, (**B**) NIH-3T3, (**C**) Neuro-2a. (**D**) Hemolysis ratio of PMC NPs in different concentrations (*n* = 3).

The cytotoxicity of PM and PMC NPs was analyzed in mouse neuroblastoma Neuro-2a, mouse fibroblast NIH-3T3, and microglia SIM-A9 cell lines via a CCK-8 assay. Both PM and PMC NPs demonstrated negligible cell toxicity after 24 h at concentrations of 2.5–20 μg/mL in the SIM-A9 cell line, with cell viability remaining at over 80% for PM NPs and over 90% for PMC NPs (Figure 4A). As shown in Figure 4B, similar results were observed in the NIH-3T3 cell line when treated with PM NPs, while PMC NPs showed no obvious cytotoxicity (≥100% cell survival). When incubated with Neuro-2a, PM NPs showed slight 24 h cytotoxicity (70–80% cell survival) at concentrations of 5–40 μg/mL, while PMC NPs demonstrated acceptable cytocompatibility (>80%) (Figure 4C). Moreover, data from the hemolysis assay indicated that no hemolysis occurred when treated with either type of NP at varying concentrations (2.5–80 μg/mL), reflecting favorable blood compatibility (Figure 4D). These results indicate that both PM and PMC NPs possess acceptable biocompatibility, potentially ensuring their suitability for subsequent biomedical applications.

### 3.4. PMC NPs Induces an Anti-Inflammatory Response in Microglia

**Figure 5:**
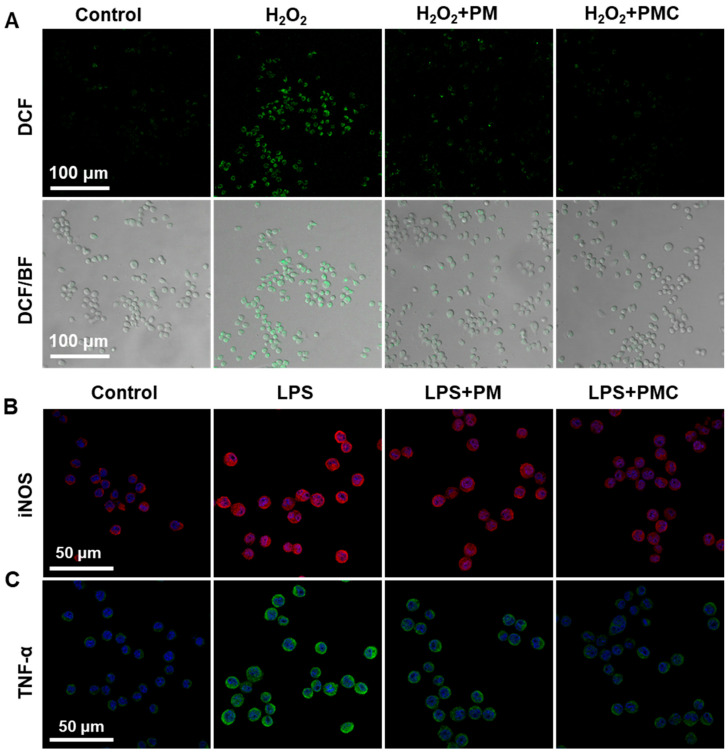
Immunofluorescence of ROS and inflammatory cytokines in microglia. (**A**) Fluorescence images of total intracellular ROS in SIM-A9 after different treatments for 4 h. (**B**) The iNOS and (**C**) TNF-α immunofluorescence images of SIM-A9 cultured with PM and PMC NPs in LPS condition (concentration of H_2_O_2_: 200 μM, LPS: 200 ng/mL, PM and PMC NPs: 10 μg/mL).

Microglia are the resident immune cells of the CNS, and they play a key role in various inflammatory-related degenerative and traumatic CNS diseases, such as AD, PD, TBI, and SCI. Excessive ROS is an upstream cue of inflammation mediated by microglia in the CNS, generally serving as a trigger of inflammation responses [34,35]. Therefore, the ROS clearing conducted by PM and PMC NPs in microglia was first tested using a DCF probe. As presented in Figure 5A, a strong ROS signal was observed in microglia induced by H_2_O_2_, while this signal was significantly weaker after treatment with PM and PMC NPs. Furthermore, the PMC NP-treated microglia showed a lower ROS level compared to PM NPs, corresponding with the control group. These data suggest that PMC NPs possess effective ROS scavenging ability in microglia, which might also regulate downstream inflammation.

**Figure 6:**
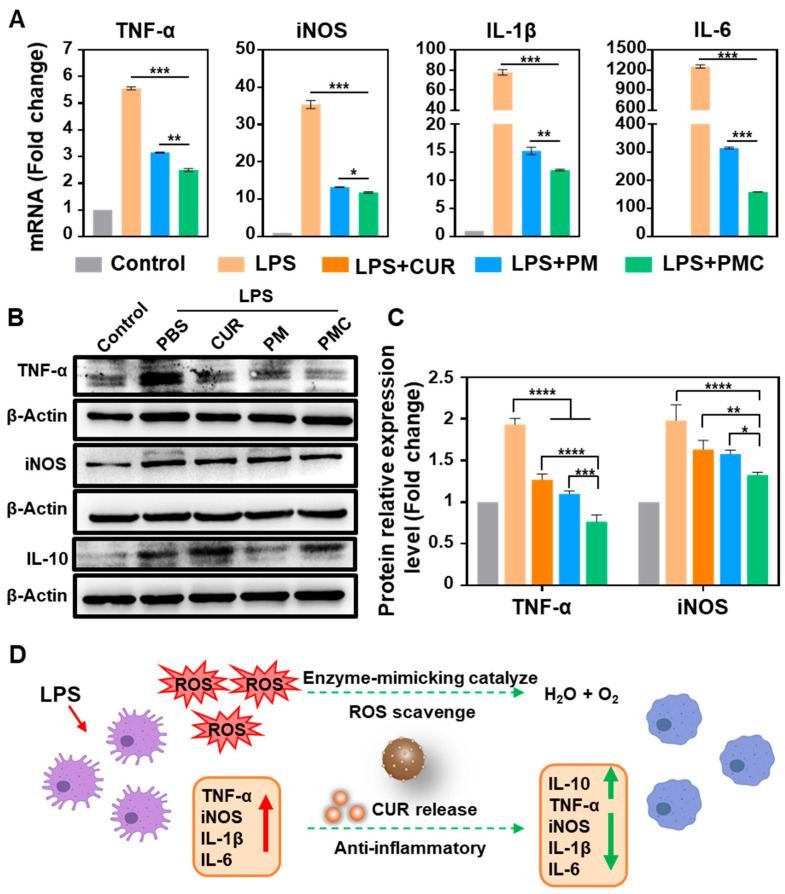
Anti-inflammatory ability of PMC NPs in microglia. (**A**) The mRNA expression levels of TNF-α, iNOS, IL-1β, and IL-6 in SIM-A9. (**B**,**C**) The protein expression of TNF-α, iNOS, and IL-10 in SIM-A9 by Western blot analysis. (**D**) Schematic presentation of antioxidative and anti-inflammation activities of PMC NPs. A one-way ANOVA test of multiple comparisons followed by Dunnett’s post hoc test was used in all analyses. * *p* < 0.05, ** *p* < 0.01, *** *p* < 0.001, **** *p* < 0.0001 (*n* = 3).

Then, we assessed the anti-inflammatory ability of PM and PMC NPs in microglia. First, immunofluorescence images showed that treatment with PMC NPs could significantly inhibit the increased expression of classically pro-inflammatory cytokines TNF-α and iNOS induced by LPS (Figure 5B,C). Real-time fluorescent quantitative PCR (qRT-PCR) was conducted to test the expression of pro-inflammatory cytokines at the mRNA level. LPS-treated SIM-A9 significantly increased the expression of TNF-α, iNOS, IL-6, and IL-1β. Subsequently, treatment with both PM and PMC NPs in active microglia resulted in a significant decrease in the expression of all four genes (Figure 6A). We further studied the expression of TNF-α, iNOS, and IL-10 at the protein level using Western blot. The results confirmed the anti-inflammatory capability of PMC NPs showing that pro-inflammatory cytokines TNF-α and iNOS were downregulated and anti-inflammatory cytokine IL-10 was upregulated (Figure 6B,C). Among all the immunofluorescence, qRT-PCR, and western blot assays, PMC NPs exhibited a better anti-inflammatory effect than PM NPs and curcumin. This suggests that the decoration of curcumin in the nanohybrid played a critical role in the potential therapeutic effect. PM NPs may have a synergistic effect in scavenging ROS and relieving inflammation. These results suggest that PMC NPs could effectively relieve oxidative stress and inhibit the classical pro-inflammatory activation of microglia (Figure 6D).

### 3.5. PMC NPs Induce an Antioxidative Response and Cytoprotection

**Figure 7:**
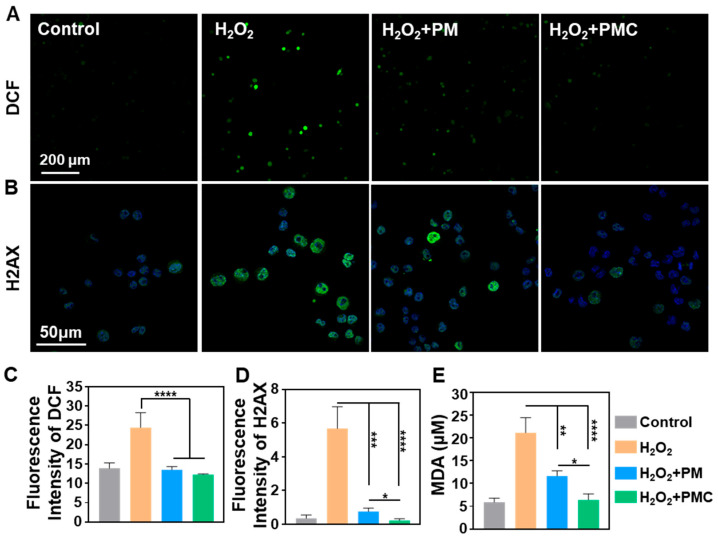
Antioxidant ability of PMC NPs in Neuro-2a. (**A**) Fluorescence images and (**C**) quantification of total intracellular ROS in Neuro-2a after different treatments for 4 h. (**B**) The H2AX immunofluorescence images and (**D**) quantification of Neuro-2a cultured with PM and PMC NPs in H_2_O_2_ condition. (**E**) MDA levels in Neuro-2a after different treatments for 6 h (concentration of H_2_O_2_: 200 μM, PM and PMC NPs: 10 μg/mL). A one-way ANOVA test of multiple comparisons followed by Dunnett’s post hoc test was used in all analyses. * *p* < 0.05, ** *p* < 0.01, *** *p* < 0.001, **** *p* < 0.0001 (*n* = 3).

The antioxidant and cytoprotection functions of PM and PMC NPs were further investigated in Neuro-2a cells, which were used to mimic neurons for this part of the experiment. The intracellular ROS level induced by H_2_O_2_ was significantly decreased in the groups treated with PM and PMC NPs, as indicated by DCF staining (Figure 7A,C). The oxidative DNA damage marker, H2AX, was effectively reduced as a result of the treatment with PM and PMC NPs in Neuro-2a cells, and PMC NPs showed an enhanced effect (Figure 7B,D). Meanwhile, the lipid peroxidation level in the oxidatively damaged Neuro-2a was evaluated using the MDA concentration. As shown in Figure 7E, both PM and PMC NPs could effectively reduce the MDA concentration in Neuro-2a. Similar to the oxidative DNA damage results, PMC NPs possessed a stronger antioxidative ability intracellularly, which may be attributed to the additive effect of PM NPs and loaded curcumin. Since oxidative damage triggers secondary damage and chronic inflammation in various nervous diseases, these demonstrated antioxidant properties suggest the promising application of PMC NPs in nerve tissue repair.

**Figure 8:**
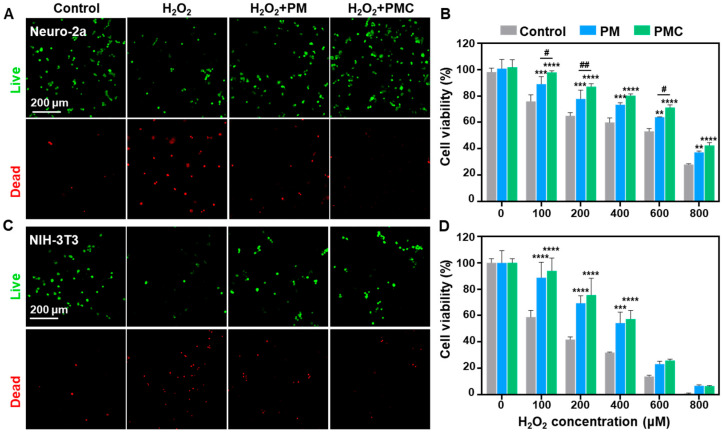
Cytoprotection of PM and PMC NPs. (**A**) Fluorescence images of live/dead (green/red) staining and (**B**) cell viability of Neuro-2a treated with 10 μg/mL PMC NPs with 200 μM H_2_O_2_ for 6 h. (**C**) Fluorescence images of live/dead (green/red) staining and (**D**) cell viability of NIH-3T3 treated with 10 μg/mL PMC NPs with 200 μM H_2_O_2_ for 6 h. A two-way ANOVA test of multiple comparisons was used in all analyses. ** *p* < 0.01, *** *p* < 0.001, **** *p* < 0.0001 vs. Control group; # *p* < 0.05, ## *p* < 0.01, represent PM group vs. PMC group (*n* = 3).

To further investigate the cytoprotection effect of PMC NPs, we constructed an H_2_O_2_ induced oxidative damage model in Neuro-2a and NIH-3T3 cells and studied cell viability by live/dead staining and the CCK-8 assay. According to the results in Figure 8A,B, both PM and PMC NPs could rescue the cells from oxidative damage in Neuro-2a, and cell viability was significantly improved at different H_2_O_2_ concentrations with treatment. Moreover, cells incubated with PMC NPs exhibited higher viability, even maintaining 80% cell viability at an H_2_O_2_ concentration of 400 μM. Similar results were observed in NIH-3T3 cells (Figure 8C,D), where PMC NPs could protect cells from oxidative damage, although there was a lower cell ability than in Neuro-2a under the same H_2_O_2_ concentration. This result might be explained by the fact that NIH-3T3 cells are more vulnerable to oxidative damage. Thus, these results indicated that PMC NPs can protect cells from oxidative damage and maintain satisfied cell viability.

## 4. Conclusions

In this study, a novel PMC NP nanocomposite was synthesized in a simple and cost-effective manner, demonstrating good biocompatibility. The PMC NPs exhibit specific SOD-like and CAT-like activities in physiological conditions, which can be attributed to the Mn^3+^/Mn^2+^ redox couples and loaded curcumin. Owing to the synergistic abilities of the Mn^3+^/Mn^2+^ redox couples and curcumin in ROS clearing and inhibiting pro-inflammatory cytokines, PMC NPs significantly alleviated intracellular oxidative stress and decreased the expression of inflammatory cytokines without adverse side effects in the microglia. Furthermore, PMC NPs scavenged intracellular RONS from Neuro-2a cells, reducing oxidative damage to DNA and lipids, thereby promoting cell survival in an environment with oxidative stress. In summary, our work provides a promising strategy for designing antioxidant and anti-inflammatory nanoplatforms to reprogram the complicated microenvironment in neuroinflammation-associated diseases.

## Data Availability

The datasets used during and/or analyzed during the current study are available from the corresponding author upon reasonable request.
